# Association between Arrhythmia and Pulmonary Artery Pressure in Heart Failure Patients Implanted with a Cardiac Defibrillator and Ambulatory Pulmonary Artery Pressure Sensor

**DOI:** 10.19102/icrm.2019.100903

**Published:** 2019-09-15

**Authors:** Rahul N. Doshi, Steven Carlson, Rahul Agarwal, Rupinder Bharmi, Philip B. Adamson

**Affiliations:** ^1^University of Southern California, Los Angeles, CA, USA; ^2^Abbott Laboratories, Sylmar, CA, USA

**Keywords:** Heart failure, implantable cardiac defibrillator, pulmonary artery pressure sensor, ventricular fibrillation, ventricular tachycardia

## Abstract

The association between ventricular arrhythmia (VA) burden or defibrillator therapy and pulmonary artery pressure (PAP) has not been characterized in an ambulatory setting; thus, we sought in the present research to determine the relationship between ambulatory PAP and VA burden. A retrospective cohort study involving patients with an implantable cardiac defibrillator and CardioMEMS™ PAP sensor (Abbott Laboratories, Chicago, IL, USA) both transmitting remotely into the Merlin.net™ patient care network (Abbott Laboratories, Chicago, IL, USA) was conducted. VA and therapy burden in the six months following sensor implant were stratified by the baseline mean PAP. Patients with PAPs of 25 mmHg to 35 mmHg and those with PAPs of 35 mmHg or more were compared with individuals with PAPs of less than 25 mmHg. The change in VA burden was reported using the averaged mean PAP reduction during the first three months. A total of 162 patients aged 69.4 years ± 10.9 years were included (74% male) with a baseline mean PAP of 36.2 mmHg ± 10.4 mmHg. Twenty patients with a baseline mean PAP of less than 25 mmHg had no VAs over six months. For 61 patients with a baseline mean PAP of between 25 mmHg and 35 mmHg, the annualized number of days with ventricular tachycardia (VT)/ventricular fibrillation (VF) was 1.65/patient-year (p < 0.001), with 8% of patients having VT/VF events. For 81 patients with a baseline mean PAP of 35 mmHg or more, 19% of patients had a VT/VF event and an annualized number of days with VT/VF events of 1.45/patient-year (p < 0.001). When analyzing the treatment effect, a reduction of 3 mmHg or more in mean PAP over three months reduced arrhythmia burden over the next three months as compared with in patients without such an improvement. In conclusion, it is indicated that VAs are associated with high PAPs, and a reduction in PAP may lead to a reduction in VAs in real-world ambulatory patients.

## Introduction

Ventricular arrhythmias (VAs) are highly prevalent in patients with advanced heart failure (HF).^1^ Elevated intracardiac pressures may provoke the onset of VAs. Acute ventricular dilatation in animal preparations have been shown to have arrhythmogenic effects.^2^ The average daily median estimated pulmonary artery diastolic (ePAD) pressure has been shown to be associated with an increased risk for ventricular tachycardia (VT)/ventricular fibrillation (VF).^3^ In the Reducing Decompensation Events Utilizing Intracardiac Pressures in Patients with Chronic HF (REDUCEhf) trial, the absolute value of the ePAD pressure measurement was not found to be associated with arrhythmic risk. However, there was no decrease in the primary endpoint of HF events,^4^ perhaps due to an insufficient level of specificity in the measurement.

Treatment of ambulatory hemodynamic monitoring with a pulmonary artery pressure (PAP) sensor has been shown to lower HF hospitalizations and PAP in the real world^5,6^ as well as in the CardioMEMS™ Heart Sensor Allows Monitoring of Pressure to Improve Outcomes in New York Heart Association Class III HF Patients (CHAMPION) clinical trial.^7^ However, the association of VAs and PAP has not been studied in an ambulatory setting to date to our knowledge. Thus, the purpose of this study was to characterize the association between baseline PAP and VA and to evaluate the hypothesis that a reduction in PAP leads to a reduction in VT/VF.

## Methods

In order to answer the aforementioned questions, we conducted a retrospective cohort study among patients who had received implantable cardioverter-defibrillators (ICDs; Abbott Laboratories, Chicago, IL, USA) and an ambulatory PAP sensor (CardioMEMS™; Abbott Laboratories, Chicago, IL, USA) between August 2014 and March 2016.

### Patients

The study cohort was derived using Abbott Laboratories’ proprietary patient device tracking system and the Merlin.net™ (Abbott Laboratories, Chicago, IL, USA) database to retrieve patient device type, patient characteristics (age and gender), ICD device session records, and PAP transmissions. The study cohort consisted of patients with a prior ICD who were then implanted with a PAP sensor (CardioMEMS™; Abbott Laboratories, Chicago IL, USA) between August 2014 and March 2016. Of these, patients with missing age, gender, implant date, or baseline PAP data and/or any absence of overlapping transmissions between their ICD and PAP sensor were excluded. PAP sensor implant was used as the index event, and patients were followed up with to either six months later or the time of the last transmission from the PAP sensor, whichever came earlier.

### Pulmonary artery pressure analysis

Patients with the implanted pressure sensor routinely transmitted their PAP measurements at around the same time daily. In cases having multiple transmissions per day, the PAP values were averaged to create a single PAP measurement for the day. When there were no transmissions on a given day, linear interpolation was used for imputation. The average of PAP measurements across the first seven days after PAP sensor implant was used as the baseline value. The area under the curve (AUC)^6^ was then computed as an integral of the relative difference between pressure measurements.

### Programming, ventricular tachycardia/ventricular fibrillation events, and therapy analysis

Session records from the ICD devices were used to retrieve the programming, VT/VF event history, and all related device therapy [ie, antitachycardia pacing (ATP) and shocks] data. Using these session records, we determined the number of arrhythmia events (VT/VF) and number of days with events over the time period spanning from the date of sensor implant through the study follow-up period. Annualized event rates and days with events were then calculated by dividing by the number of days with valid transmissions in the record. For evaluating device programming, the number of zones programmed, the minimum ventricular rate for the detection of VT and VF, and the number of R–R intervals waited out before flagging VT or VF detection were retrieved at the time of sensor implant and at three months following sensor implant, respectively.

### Relation between pulmonary artery pressure and arrhythmia

To study the relation between PAP and arrhythmia, we looked at the rates of all VT/VF events, ATP events, and shocks in patients with baseline mean PAPs of less than 25 mmHg (group 1), 25 mmHg to 35 mmHg (group 2), and 35 mmHg or more (group 3), respectively. To determine if this was driven by multiple events in the same day, we also looked at the rate of days with VT/VF events, ATP events, and shocks as well as the number of patients with the same in the aforementioned three groups.

### Relation between change in pulmonary artery pressure and arrhythmia

Separately, to study the effects of changes in PAP on arrhythmia burden, two groups were formed having AUCs for mean pressures of less than −270 mmHg or −270 mmHg or more, respectively, at three months. The AUC value of 270 mmHg corresponds to a cumulative decrease of 3 mmHg during the first three months after sensor implant. Subsequently, the number of patients with events, number of days with events, and the rate of days with events were examined for between zero months and three months (period 1) and between three months and six months (period 2) after sensor implant, respectively.

### Statistical analysis

Results are presented as means ± standard deviations. Further, p-values were computed using a t-test for continuous variables, the chi-squared test for independence for categorical variables, and the bootstrap method for event rates, unless otherwise stated. All analyses were conducted using the Python programming language (Python Language reference version 3.5.3; Python Software Foundation, Wilmington, DE, USA)) and R version 3.1.1 (The R Foundation, Vienna, Austria).

All data retrieved for analysis were retrospective and deidentified. Consultation with our local institutional review board (IRB) confirmed no need for IRB approval; similarly, the need for patient consent was also waived.

## Results

**Figure 1:**
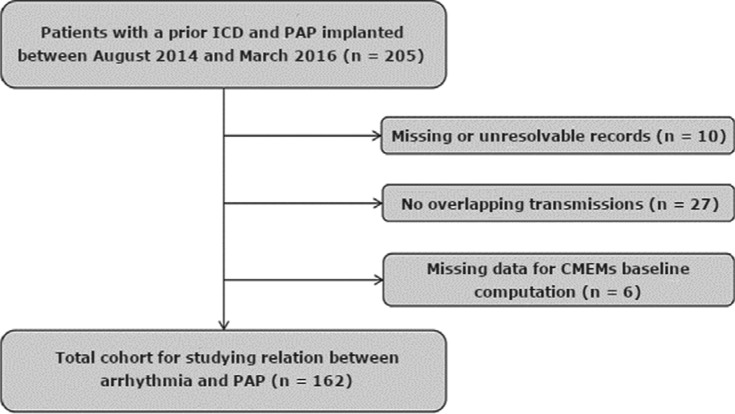
CONSORT diagram describing the study population derivation.

We identified 205 ICD implanted patients who were then implanted with a PAP sensor between August 2014 and March 2016. Among these, 10 patients had either missing records (eg, age, gender, date of implant) or an unresolvable record. Twenty-seven did not have any overlapping PAP transmissions and ICD session recordings. Six patients had fewer than seven days of follow-up, which was the minimum necessary for the computation of baseline PAP. After excluding these patients for the aforementioned reasons, our final study cohort consisted of 162 patients **(Figure 1)**. This cohort was aged 69.4 years ± 10.9 years and was 74% male, with 41% being ICD recipients and 59% being cardiac resynchronization therapy defibrillator (CRT-D) recipients. The average follow-up time over the study period was 0.46 years ± 0.11 years.

**Figure 2:**
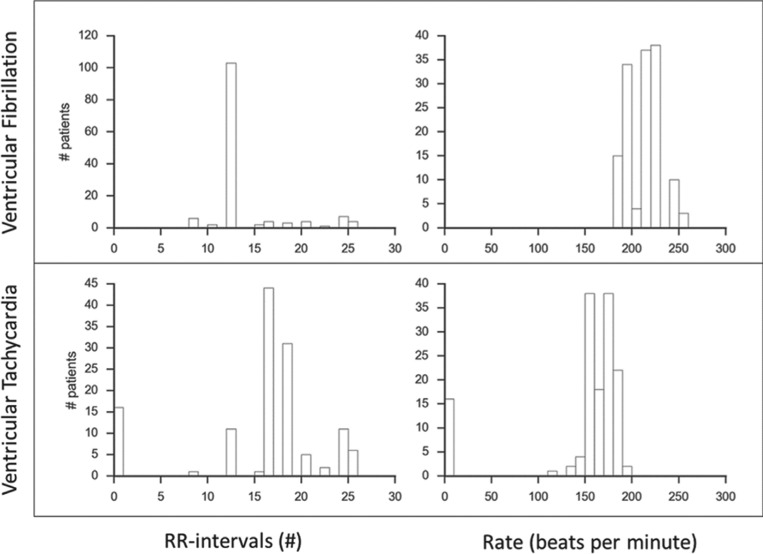
ICD programming at sensor implant. VF and VT detection programming: rate cutoff (beats per minute) and number of R-R intervals (n).

The device interrogations records with therapy programming were available in 141 of the 162 patients. The arrhythmia detection programming was as follows: 16 (11%) patients were programmed with one VF zone, 78 (55%) patients were programmed with one VF zone and one VT zone, and 47 (34%) patients were programmed with one VF zone and two VT zones. **Figure 2** shows the number of R–R intervals used for detection and ventricular rate cutoffs for VF and VT detection at sensor implant. In most devices, VF detection was set at 12 intervals and a range of 190 bpm to 230 bpm, whereas VT detection was set at 16 to 18 intervals and a range of 150 bpm to 190 bpm.

### Relation between pulmonary artery pressure and arrhythmia

The association of PAP and arrhythmia was studied in patients stratified by their baseline mean PAP. **Table 1** shows the demographic characteristics of patients in groups 1, 2, and 3. There was no significant difference in age or device type among the three groups; however, there were significantly more males (84% versus 45%) in group 3 as compared with in group 1. The arrhythmia detection and ICD therapy programming were not different between the three groups.

Among patients in group 1 (n = 20), there were no VT/VF events for the full duration of follow-up. Among patients in group 2 (n = 61), there were 148 VT/VF episodes that occurred over 45 unique patient-days in five patients. These VAs were treated via 181 ATP applications and seven shocks. The 148 episodes occurred over 45 days with zero median events/patient and two patients having event-rates of 68 events/year and 216 events/year, respectively.

In group 3 (n = 81), 13 patients experienced 438 VT/VF episodes that occurred over 53 days. These arrhythmias were treated via 471 ATP applications and 32 shocks. The 438 episodes occurred over 53 days with zero median events/patient and one patient having an event rate of 770 events/year.

**Figure 3:**
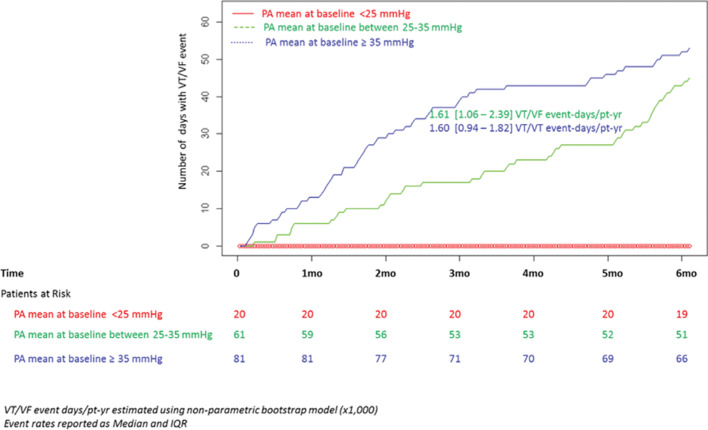
Temporal trends in baseline PAP and VT/VF burden.

**Figure 3** depicts the accumulation of days with episodes over the six-month follow-up for the three groups. Using the nonparametric bootstrap method, the median VT/VF event-days/patient-year value in group 3 was 1.60 (interquartile range: 0.94–1.82), while that in group 2 was 1.61 (interquartile range: 1.06–2.39). While these event rates were no different statistically (hazard ratio: 0.84; p = 0.71) from each other, both values were significantly higher than that for subjects in group 1 (p < 0.001).

### Relation between change in pulmonary artery pressure and arrhythmia

**Table 2** shows demographic characteristics of patients by AUC according to improved AUC (< −270 mmHg) and worsened AUC (≥ −270 mmHg) at three months. There were 62 patients and 82 patients in the two groups, respectively, with a follow-up period after PAP sensor implant of close to six months. The mean age in each AUC group was approximately 70 years old, and there were no significant differences in gender or device type distribution. The arrhythmia detection and ICD therapy programming were additionally not different between the two groups. Further, the number of patients with programming changes in the first three months and the magnitude of programming changes were also not significantly different between these two groups (changes in VF interval to detect: 0 versus 0.25, p = 0.4 and VF detection rate: 0.27 versus 0 bpm, p = 0.24; VT interval to detect: 0.35 versus 0.08, p = 0.3 and VT detection rate: 0.09 versus 0.22 bpm, p = 0.62). Furthermore, there was no significant difference in programming between the patients who had VT/VF events occurring between three and six months after sensor implant and those who did not (VF interval to detect: 13 versus 14, p = 0.56 and VF detection rate: 221 versus 207 bpm, p = 0.24; VT interval to detect: 18 versus 18, p = 0.97 and VT detection rate: 173 versus 141 bpm, p = 0.12).

**Figure 4:**
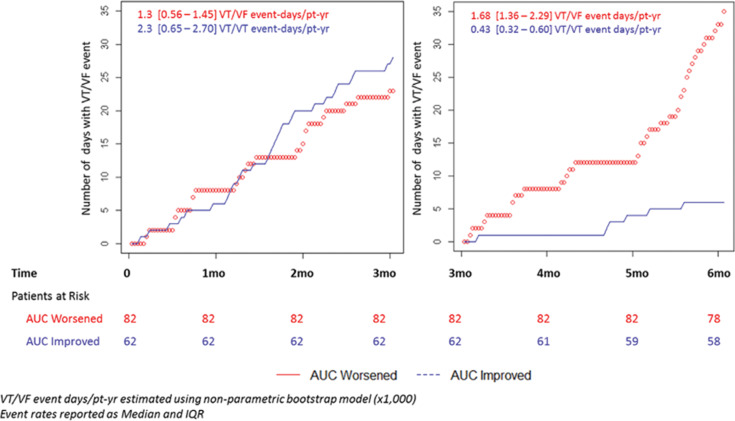
Association of changes in PAP and VT/VF burden.

**Figure 4** depicts the accumulation of days with episodes in the two AUC groups during periods 1 and 2 (used to assess the PAP improvement and used to assess the association of PAP reduction and VT/VF burden, respectively). In the assessment period, the AUC-worsened group had 23 VT/VF event-days, whereas the AUC-improved group had 28 T/VF event-days, with event rates of 1.3 (0.56–1.45) VT/VF event-days/patient-year and 2.3 (0.65–2.70) VT/VT event-days/patient-year, respectively. Over the next three months, the AUC-worsened group continued to have a high number of episodes with 1.68 (1.36–2.29) VT/VF event days/patient-year, whereas the AUC-improved group had a reduction in episodes, resulting in an outcome of 0.43 (0.32–0.60) VT/VF event days/patient-year. A comparison of the AUC-worsened and AUC-improved groups in period 1 demonstrated an incidence rate ratio (IRR) of 1.75 (0.53–2.19; p = 0.72) and the same comparison in period 2 demonstrated an IRR of 0.25 (0.16–0.37; p = 0.035).

## Discussion

To our knowledge, this is the first demonstration of the idea that ambulatory PAP as determined by an implantable PAP sensor has a positive correlation with the risk of VAs in a population with cardiomyopathy and symptomatic HF who have received an ICD or CRT-D implant. In addition, this is the first demonstration that, in a real-world cohort, a favorable response to treatment presumably using ambulatory PAP recordings correlates with a decreased risk of VA.

We demonstrated that patients with PAPs of less than 25 mmHg have a reduced rate of VAs, while patients with elevated PAPs have a threefold increase in their likelihood for VAs, with the difference being highly statistically significant. This is in sharp contrast to the data calculated utilizing the ePAD measurement in the REDUCEhf trial.^2^ Thus, our data suggest a positive correlation between the risk of VT/VF and HF exacerbation.^8^ This might be explained by the increased specificity of the pressure measurement from the PAP sensor as compared with ePAD. This would be in keeping with the concept that acute ventricular dilatation results in both a failing heart and increased arrhythmogenic risk. In addition, this may suggest treatment be tailored to achieve a target goal as compared with simply reducing elevated PAP.

Similarly, the data presented here show an increased number of days with VA events associated with elevated PAPs when compared with normal measurements, which also reached statistical significance. Reiter et al. previously demonstrated this trend with ePAD measurements, but their investigation failed to reach statistical significance.^2^ This suggests an increased sensitivity of the PAP sensor in detecting changes that result in an increased risk of VT/VF when compared with the ePAD device.

Finally, the data presented here demonstrate that a favorable reduction in targeted PAP reduction results in a decreased rate of VA in an otherwise-matched cohort. Patients in the less than −270 mmHg AUC group corresponding to an average reduction of 3 mmHg of mean PAP had a significantly lower event rate for VAs when compared with the group with lesser or no improvement. This suggests that there is a treatment effect that not only reduces the risk of HF hospitalization as previously reported but also impacts the frequency of VA events. To our knowledge, this is the first demonstration that successful ambulatory HF treatment decreases the likelihood of VA in an ICD/CRT-D population. This decreased risk is consistent with other treatments for HF, including medical therapy^9,10^ and CRT.^11,12^

The mechanism by which elevated pulmonary pressures may increase susceptibility to VAs is likely multifactorial. Ventricular stretch is associated with shortened action-potential duration and repolarization.^2^ HF exacerbation is also associated with increased sympathetic tone in this population.^13^ In the CHAMPION trial, the use of a PAP sensor resulted in increased dosages of diuretics, vasodilators, and neurohormonal antagonists^14^ and could provide an explanation for the decreased event rates. It should be recognized that the decrease in the risk of VAs demonstrated is from only a short follow-up period of six months. However, the benefits of targeted HF therapy using the implantable PAP sensor have been demonstrated for up to 18 months.^7^ Whether long-term sensor-directed treatment results in long-term arrhythmia reduction or not is yet to be demonstrated.

### Limitations

The retrospective study design of this research is a significant limitation, as the study findings were not prospectively defined. However, the data are from a real-world cohort, which limits potential biases. Separately, the cohort size is small, especially in the low-PAP group. Despite the small sample size, the results reached statistical significance. The arrhythmia events were determined based on device characterization from the remote monitoring system and individual episodes were not independently adjudicated. Thus, inappropriate therapy could not be excluded. As with other studies evaluating VAs or ICD therapy, a small number of patients could contribute to a large number of events secondary to arrhythmia storm. However, our data show both a decrease in the number of events and the number of days at risk for VT/VF associated with a lower PAP, and there was no significant change in device programming. Finally, the data were sourced from a deidentified dataset from a remote monitoring system and thus individual patient characteristics including the extent of disease and medications could not be compared, therefore limiting the ability to compare patient groups.

### Future directions

This study demonstrates that data from ambulatory hemodynamic monitoring can be coupled with data from ICD remote monitoring, obtained from the same Internet portal. Potential hemodynamic measurements obtained via the ICD implanted could lead to greater fidelity in determining the relationship between pressures and arrhythmias, as well as potentially establishing an assessment of the hemodynamic stability of any given arrhythmia.

## Conclusions

In this real-world cohort of ICD recipients who received a PAP sensor, a high baseline pressure was associated with a high burden of VAs. PAP sensor–guided reduction in mean PAP in patients with symptomatic HF was associated with a reduction in arrhythmia burden. Our preliminary analysis warrants prospectively studying the relation of change in PAP with the change in arrhythmia in a larger cohort and whether targeting an absolute target value significantly reduces the risk of ICD therapy. HF patients with multiple devices may be uniquely suited for management based on combined information to reduce the risk of arrhythmia while improving HF status.

## Figures and Tables

**Table 1:** Patient Demographics Stratified by Mean PAP at Baseline.

	Group 1 (< 25 mmHg) Mean PAP at Baseline	Group 2 (25–35 mmHg) Mean PAP at Baseline	p-value*	Group 3 (≥ 35 mmHg) Mean PAP at Baseline	p-value*
Number of patients	20 patients	61 patients		81 patients	
Follow-up	0.5 ± 0.0 years	0.45 ± 0.1 years		0.45 ± 0.1 years	
Age	67.5 ± 10.8 years	70.4 ± 11.4 years	0.325	69.2 ± 10.7 years	0.545
Male gender	9 (45%) patients	42 (69%) patients	0.099	68 (84%) patients	< 0.001
CIED					
ICD only	11 (55%) patients	23 (38%) patients	0.271	33 (41%) patients	0.368
CRT-D	9 (45%) patients	38 (62%) patients		48 (59%) patients	
Programming**					
VF interval to detect***	13.6 ± 5.3	13.6 ± 4.5	0.990	14.3 ± 5.1	0.598
VF detection rate	214.4 ± 18.3	211.1 ± 14.7	0.437	213.2 ± 15.1	0.768
VT interval to detect***	15.4 ± 7.9	17.4 ± 9.1	0.430	18.4 ± 11.2	0.300
VT detection rate	129.4 ± 61.0	146.57 ± 50.7	0.246	149.6 ± 55.0	0.177

CIED: cardiac implantable electronic device; ICD: implantable cardioverter-defibrillator; CRT-D: cardiac resynchronization therapy defibrillator; VF: ventricular fibrillation; VT: ventricular tachycardia.

*As compared with mean PAP at baseline in the less than 25 mmHg group.

**Programming data available for 18, 51, and 72 patients in the three groups, respectively.

***Number of ventricular beats the device waits before definitively identifying the arrhythmia.

**Table 2:** Patient Demographics Stratified by Improvement in PAP at Three Months.

	AUC < −270 mmHg	AUC ≥ −270 mmHg	p-value
Number of patients	62 patients	82 patients	
Follow-up	0.24 ± 0.04 years	0.25 ± 0.01 years	
Age	69.5 ± 10.7 years	69.7 ± 10.4 years	0.898
Male gender	43 (69%) patients	62 (76%) patients	0.518
Mean PAP at baseline	39.0 ± 10.2 mmHg	33.5 ± 9.6 mmHg	0.001
CIED			
ICD only	30 (48%) patients	32 (39%) patients	0.340
CRT-D	32 (52%)	50 (61%)	
Programming at three months			
VF interval to detect	13.9 ± 4.9	14.4 ± 5.6	0.613
VF detection rate	212.2 ± 15.8	212.8 ± 16.1	0.836
VT interval to detect	17.1 ± 8.7	18.2 ± 11.4	0.547
VT detection rate	142.4 ± 57.4	147.5 ± 54.0	0.608
Patients with programming changes in the first three months*	2 (3.8%)	2 (2.7%)	0.866

CIED: cardiac implantable electronic device; ICD: implantable cardioverter-defibrillator; CRT-D: cardiac resynchronization therapy defibrillator; VF: ventricular fibrillation; VT: ventricular tachycardia.

*Programming data available for review from 52/62 and 73/82 patients, respectively.
